# Intravenous Arginine Stimulates Glucagon Secretion More Than Equimolar Alanine, Leucine, Glutamine, and Proline in Humans

**DOI:** 10.1210/jendso/bvaf139

**Published:** 2025-08-23

**Authors:** Malte Palm Suppli, Astrid Høgedal, Jonatan Ising Bagger, Magnus Frederik Gluud Grøndahl, Julie Lyng Forman, Samuel Addison Jack Trammell, Ana Vranešević, Trisha Jean Grevengoed, Hendrik Vilstrup, Mikkel Bring Christensen, Nicolai Jacob Wewer Albrechtsen, Asger Bach Lund, Jens Juul Holst, Filip Krag Knop

**Affiliations:** Center for Clinical Metabolic Research, Gentofte Hospital, University of Copenhagen, DK-2900 Hellerup, Denmark; Center for Clinical Metabolic Research, Gentofte Hospital, University of Copenhagen, DK-2900 Hellerup, Denmark; Center for Clinical Metabolic Research, Gentofte Hospital, University of Copenhagen, DK-2900 Hellerup, Denmark; Department of Diabetes Care, Steno Diabetes Center Copenhagen, DK-2730 Herlev, Denmark; Center for Clinical Metabolic Research, Gentofte Hospital, University of Copenhagen, DK-2900 Hellerup, Denmark; Section of Biostatistics, Department of Public Health, University of Copenhagen, DK-1353 Copenhagen, Denmark; Department of Biomedical Sciences, Faculty of Health and Medical Sciences, University of Copenhagen, DK-2200 Copenhagen, Denmark; Department of Biomedical Sciences, Faculty of Health and Medical Sciences, University of Copenhagen, DK-2200 Copenhagen, Denmark; Department of Biomedical Sciences, Faculty of Health and Medical Sciences, University of Copenhagen, DK-2200 Copenhagen, Denmark; Department of Hepatology and Gastroenterology, Department of Clinical Medicine, Aarhus University, DK-8200 Aarhus, Denmark; Center for Clinical Metabolic Research, Gentofte Hospital, University of Copenhagen, DK-2900 Hellerup, Denmark; Department of Clinical Medicine, Faculty of Health and Medical Sciences, University of Copenhagen, DK-2200 Copenhagen, Denmark; Department of Clinical Pharmacology, Bispebjerg Hospital, University of Copenhagen, DK-2400 Copenhagen, Denmark; Department of Clinical Medicine, Faculty of Health and Medical Sciences, University of Copenhagen, DK-2200 Copenhagen, Denmark; Copenhagen Center for Translational Research, Bispebjerg Hospital, University of Copenhagen, DK-2400 Copenhagen, Denmark; Novo Nordisk Foundation Center for Protein Research, Faculty of Health and Medical Sciences, University of Copenhagen, DK-2200 Copenhagen, Denmark; Department of Clinical Biochemistry, Copenhagen University Hospital—Bispebjerg, DK-2400 Copenhagen, Denmark; Center for Clinical Metabolic Research, Gentofte Hospital, University of Copenhagen, DK-2900 Hellerup, Denmark; Department of Diabetes Care, Steno Diabetes Center Copenhagen, DK-2730 Herlev, Denmark; Department of Clinical Medicine, Faculty of Health and Medical Sciences, University of Copenhagen, DK-2200 Copenhagen, Denmark; Department of Biomedical Sciences, Faculty of Health and Medical Sciences, University of Copenhagen, DK-2200 Copenhagen, Denmark; Novo Nordisk Foundation Center for Basic Metabolic Research, Faculty of Health and Medical Sciences, University of Copenhagen, DK-2200 Copenhagen, Denmark; Center for Clinical Metabolic Research, Gentofte Hospital, University of Copenhagen, DK-2900 Hellerup, Denmark; Department of Clinical Medicine, Faculty of Health and Medical Sciences, University of Copenhagen, DK-2200 Copenhagen, Denmark; Medical and Translational Science, Novo Nordisk A/S, DK-2880 Bagsværd, Denmark

**Keywords:** amino acids, arginine, hepatic steatosis, liver α cell axis, metabolic dysfunction-associated steatotic liver disease

## Abstract

**Context:**

Amino acids are known to stimulate glucagon secretion, and most amino acids can elicit a glucagon response after IV administration. Recent studies have identified a feedback loop between the liver and pancreatic α cells, regulated by glucagon and circulating amino acids, termed the liver-α cell axis.

**Objective:**

We compared the glucagonotropic effects of amino acids suggested to drive the liver-α cell axis in humans.

**Methods:**

We recruited 12 healthy male participants for a double-blind, randomized study. Each participant received equimolar bolus injections of alanine, arginine, leucine, glutamine, proline, and saline (placebo) after an overnight fast on separate days.

**Results:**

Arginine significantly increased glucagon plasma concentrations compared to placebo, evaluated by the incremental area under the curve after 30 minutes ([mean ± SD] 133 ± 71 vs 34 ± 34 pmol/L × min) and the maximum concentration of glucagon after injection (44 ± 18 vs 15 ± 4 pmol/L) (*P* < .01 for both). Alanine injection resulted in a minor increase in the peak concentration of glucagon, while glutamine showed a nonsignificant trend toward increased glucagon secretion. Insulin secretion was significantly increased by injections of arginine, alanine, and glutamine, with leucine showing a nonsignificant trend.

**Conclusion:**

In the given experimental setting, arginine was identified as the most efficient stimulator of glucagon secretion. Arginine, alanine, and glutamine stimulated insulin secretion, with arginine eliciting the largest response. Our results indicate that arginine could be involved in regulating the liver-α cell axis in humans.

Amino acid-induced stimulation of glucagon secretion has been known since the 1960s [1-3]. It has turned out that most amino acids, except the branched-chain amino acids, are capable of eliciting a glucagon response [4-7]. One of the suggested physiological roles of amino acid-stimulated glucagon secretion is to prevent hypoglycemia due to the insulin rise in response to glucose and amino acids after a mixed meal [8]. Additionally, glucagon promotes hepatic amino acid metabolism, including the stimulation of ureagenesis to remove ammonia resulting from the transamination of amino acids [9]. Research over the past decades researchers have outlined a feedback cycle between the liver and pancreatic α cells, regulated by glucagon and circulating amino acids, known as the liver-α cell axis [10-13]. The development of obesity and hepatic steatosis is thought to disrupt the liver-α cell axis, leading to resistance toward glucagon's effect on amino acid catabolism [13]. This results in increased circulating amino acids, leading to hyperglucagonemia, which contributes to the hyperglycemia of patients with type 2 diabetes [12]. This has sparked renewed interest in investigating the glucagonotropic effects of amino acids to identify potential drivers of the liver-α cell axis, with alanine and glutamine proposed as potential candidates [14-16].

Arginine is often referred to as the most glucagonotropic amino acid [17], and its ability to stimulate both insulin and glucagon secretion is utilized in the arginine test to assess pancreatic α and β cell function [18-20]. While the ability of arginine to stimulate glucagon secretion is well documented, its postulated status as the most glucagonotropic amino acid in humans is based on scarce data [3, 21, 22]. Besides arginine, alanine is also recognized as a potent glucagon secretagogue [23, 24]. The glucagonotropic effects of amino acids have been systematically tested in various models, including dogs [4], sheep [5], and the perfused rat pancreas [25]. A recent study involving isolated perfused mouse pancreas showed that alanine, arginine, proline, and cysteine most effectively stimulated glucagon secretion, whereas glutamine did not elicit a glucagon response [26]. However, glutamine was shown to promote α cell proliferation in mouse models of glucagon resistance and has therefore also been suggested as a potential driver of the liver-α cell axis [27].

In a double-blind, randomized crossover clinical trial, we investigated the glucagonotropic and insulinotropic effects of alanine, arginine, leucine, glutamine, and proline administered as equimolar bolus injections compared to saline (placebo) in a group of healthy humans. The branched-chain amino acid leucine was included as a negative control.

## Materials and Methods

### Ethical Approval

The study was approved by the Research Ethics Committee of the Capital Region of Denmark (reg. no. H-21052615), registered with Clinicaltrials.gov (ID: NCT05954923) and conducted in accordance with the latest revision of the Declaration of Helsinki. Verbal and written informed consents were obtained from all participants before inclusion in the study.

### Study Participants

We recruited 12 healthy male participants between 20 and 65 years of age with a body mass index between 18.5 and 25 kg/m^2^. Exclusion criteria included having diabetes or first-degree relatives with diabetes, known liver disease, regular use of prescription medication, use of dietary protein supplementation, fasting plasma triglycerides ≥2 mmol/L, alanine aminotransferase and/or aspartate aminotransferase >2 × upper normal reference limits (ie, >140 U/L and > 90 U/L, respectively), and signs of liver steatosis and/or fibrosis evaluated by FibroScan® (controlled attenuation parameter value >238 dB/min and/or kPa > 6.0). We chose only to include male individuals in the study to minimize variation in the data in the context of a small exploratory study [28].

### Experimental Procedures

Each participant was studied on 6 experimental days at the Center for Clinical Metabolic Research at Gentofte Hospital, University of Copenhagen, Hellerup, Denmark. The participants consumed a standardized diet for 2 days prior to each experimental day. The diet consisted of oats with milk in the morning, a rye bread sandwich for lunch, and pasta Bolognese for dinner. The experimental days were carried out after an overnight 10-hour fast that included abstinence from coffee, water, and tobacco. On experimental days, participants were placed in a hospital bed in a comfortable semirecumbent position, and a cannula was inserted into a vein on each forearm, 1 for injections and 1 for collection of blood. The latter forearm was wrapped in a heating pad (∼42 °C) to stimulate circulation and arterialize the venous blood. At time 0 minutes, a bolus of either L-alanine, L-glutamine, arginine-hydrochloride (arginine-HCl), L-leucine, L-proline, or placebo was given over the course of 30 seconds. The amino acids were given in a double-blinded, randomized order, and each bolus contained 4 mmol of the respective amino acid dissolved in 25 mL water for injection. Blood samples were collected at −15, 0, 1, 2, 4, 6, 10, 15, 30, and 60 minutes and stored on ice. For analyses of glucagon, amino acids, glucagon-like peptide 1 (GLP-1) and glucose-dependent insulinotropic polypeptide (GIP), blood was collected in chilled tubes containing EDTA and a dipeptidyl peptidase 4 inhibitor (valine-pyrrolidide, final concentration of 0.01 pmol/L). Blood samples for analyses of insulin and C-peptide were collected in tubes containing lithium-heparin. All blood samples were centrifuged at 1200*g* and 4 °C. Plasma samples for analyses of glucagon, amino acids, GLP-1, and GIP were stored at −20 °C, and plasma samples for analyses of insulin and C-peptide were stored at −80 °C until batch analysis.

### Amino Acids

L-alanine, L-glutamine, arginine-HCl (all from Kyowa Hakko Bio Co., Ltd., Tokyo, Japan), L-leucine (Amino GmbH, Frellstedt, Germany), and L-proline (Evonik, Ham, France) were ≥ 99% pure. For each amino acid, 4 mmol was dissolved in 25 mL water for injection, dispensed into glass vials under sterile conditions, and stored at room temperature until use. The amino acid dose was based on a pilot study, in which we examined the glucagonotropic effect of various doses of arginine-HCl, which is routinely used experimentally in the arginine test to asses pancreatic islet function [18-20]. In the pilot study, we injected a bolus of arginine-HCl in the same individual on 7 separate days at doses of 0.1 g, 0.5 g, 1 g, 2.5 g (infused twice), 5g, and 7.5 g (Supplemental Fig. S1 [29]) aiming at determining a dose for submaximal stimulation of glucagon. A dose of 5 g arginine-HCl is thought to result in maximum stimulation of glucagon [17], and we observed that doses down to 1 g (4.7 mmol) of arginine-HCl elicited a comparable glucagon response. Based on that observation, we chose a dose of 4 mmol for all amino acids.

### Analyses

Plasma glucose was measured bedside with a cobas® pulse system (Roche Diagnostics International Ltd., Rotkreuz, Switzerland). C-terminal-specific radioimmunoassays were used to analyze plasma concentrations of glucagon (RRID: AB_2892837), total GLP-1 (RRID: AB_2892195), and GIP (RRID: AB_2892194). Plasma concentrations of insulin and C-peptide were analyzed using sandwich electrochemiluminescence immunoassays (ADVIA, Centaur CP, Siemens Healthcare, Ballerup, Denmark). Individual amino acid concentrations were quantified using low-resolution tandem mass spectrometry, as previously described [30]. The quantification of asparagine was altered by using labeled asparagine (¹³C₄, 99%) (Cambridge Isotopes, catalog number: CLM-8699-H-0.05) as its internal standard. The single reaction transition was 137.04 > 75.96 using a cone voltage of 17 V and a collision energy of 14 eV.

### Outcome Measures

Response curves were summarized by fasting values, maximum concentration (C_max_), area under the curve, and incremental area under the curve (iAUC). Area under the curve was calculated using the trapezoid rule and iAUC by subtracting baseline, ignoring values below baseline. The primary endpoint of the study was the iAUC for glucagon during the first 30 minutes of the experiment (iAUC_30_). Secondary endpoints included the iAUC for glucagon over the total 60 minutes of the experiment (iAUC_60_) and the C_max_ of glucagon. All other outcomes were considered exploratory.

### Statistical Analysis

Given the lack of similar previous clinical investigations, we included 12 participants corresponding to double the typical sample size in animal studies, where clear differences in the glucagonotropic effect of various amino acids were observed [4, 5]. Baseline characteristics are presented with medians and interquartile ranges, while plasma concentrations are presented with means and SDs in text and means with SEs in figures. To compare the outcome measures between the infusions, we applied a linear mixed model including infusion as fixed effect and with an unstructured covariance pattern to account for repeated measurements on each study participant. Results are reported as the estimated mean difference between amino acids and placebo with 95% confidence intervals. *P-*values were adjusted for multiple testing using the method of Benjamini and Hochberg [31], which controls the false discovery rate. Secondary and exploratory analyses were adjusted separately. All study participants completed the study (no missing data). An adjusted *P*-value < .05 was considered statistically significant. All statistical analyses were performed using R version 4.3.2 [32].

## Results

### Participant Characteristics

The participants had a median age (interquartile range) of 26.5 (24-29) years, a median body mass index of 23.1 (22.7-23.7) kg/m^2^, and a median glycated hemoglobin of 30 (28-32) mmol/mol. The participants were normolipidemic and had no signs of liver disease. Clinical characteristics of the participants are presented in Table 1.

**Table 1.** Baseline characteristics

Number (n)	12
Sex (% male)	100
Age (years)	26.5 (24-29)
Weight (kg)	79.5 (74.9-84.1)
BMI (kg/m^2^)	23.1 (22.7-23.7)
Waist-hip ratio	0.81 (0.80-0.83)
ALT (U/L)	17.5 (15-23)
AST (U/L)	15 (14-21)
GGT (U/L)	18.5 (15-24)
ALP (U/L)	66.5 (61-72)
Total cholesterol (mmol/L)	4.0 (3.8-4.2)
LDL cholesterol (mmol/L)	2.2 (2.2-2.3)
HDL cholesterol (mmol/L)	1.3 (1.2-1.4)
Triglycerides (mmol/L)	0.8 (0.6-1.1)
Glucose (mmol/L)	5.1 (4.8-5.2)
C-peptide (pmol/L)	387 (368-471)
HbA1c (mmol/mol)	30 (28-32)
HOMA-IR	0.86 (0.80-1.03)
CAP (dB/m)	197.5 (170-221)
TE (kPa)	4.7 (4.0-5.3)
FIB-4 index	0.45 (0.42-0.54)

Blood samples were drawn in the fasted state. Data are presented as medians with interquartile ranges in parentheses.

Abbreviations: ALP, alkaline phosphatase; ALT, alanine aminotransferase; AST, aspartate aminotransferase; BMI, body mass index; CAP, controlled attenuation parameter; FIB-4, fibrosis 4 index.; GGT, gamma-glutamyltransferase; HbA1c, glycated hemoglobin; HDL, high-density lipoprotein; HOMA-IR, homeostatic model assessment for insulin resistance; LDL, low-density lipoprotein; TE, transient elastography.

### Side Effects

In all participants, glutamine resulted in side effects occurring within seconds after initiation of bolus injection. The side effects included nausea, epigastric pain, dizziness, lightheadedness, and gustatory sensations (taste of metal, mint, a fresh taste, and an unspecific taste sensation were reported). The side effects lasted between 30 seconds and 8 minutes and were most prominent immediately after the administration of glutamine. The injections of alanine, arginine, leucine, proline, and placebo did not elicit any side effects.

### Increased Amino Acid Concentrations After Bolus Injection

**Figure 1.**
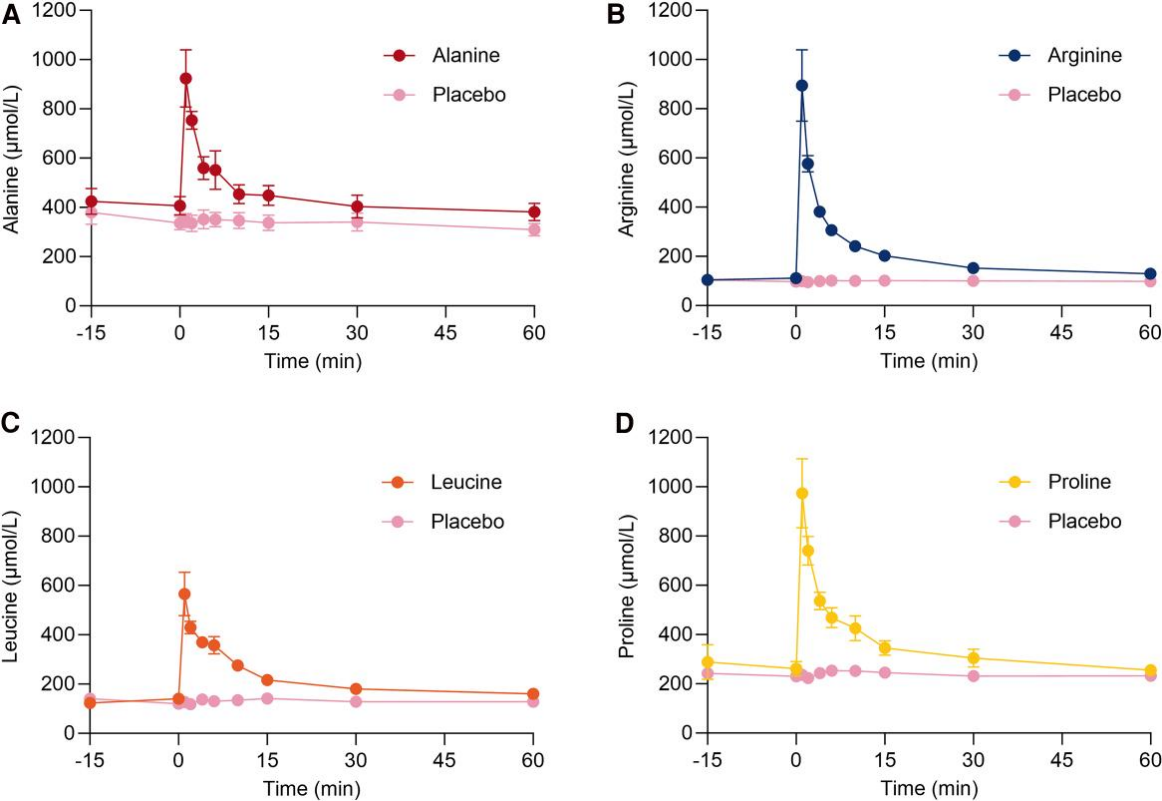
Plasma concentrations of alanine (A), arginine (B), leucine (C), and proline (D) compared to saline (placebo) after bolus injection of the respective amino acids. Injections were administered at time 0 minutes in 12 healthy male individuals on separate experimental days, conducted in a double-blind, randomized order. Data are presented as mean values ± SEM.

The plasma concentration of alanine, arginine, leucine, and proline increased immediately following bolus injection of the respective amino acids (Fig. 1). C_max_ for these amino acids was observed 1 minute postinjection: alanine [mean ± SD]: 984 ± 340 μmol/L, arginine: 937 ± 455 μmol/L, leucine: 622 ± 252 μmol/L, and proline: 1015 ± 443 μmol/L (*P* < .001 for all 4 amino acids vs placebo). The fold-change in each amino acid compared to baseline varied from 2.6 (alanine) to 8.6 (arginine) (Supplemental Table S1 [29]). We were not able to measure changes in the plasma concentration of glutamine following its injection, but mass spectrometry-based analysis of glutamine-containing vials from the same batch as those used in the experiment confirmed that they contained L-glutamine (data not shown). Bolus injection of placebo did not increase plasma amino acid concentrations. Data are presented in Fig. 1 and Supplemental Table S1 [29].

### Arginine Seems the Most Glucagonotropic Amino Acid After Bolus Injection

**Figure 2.**
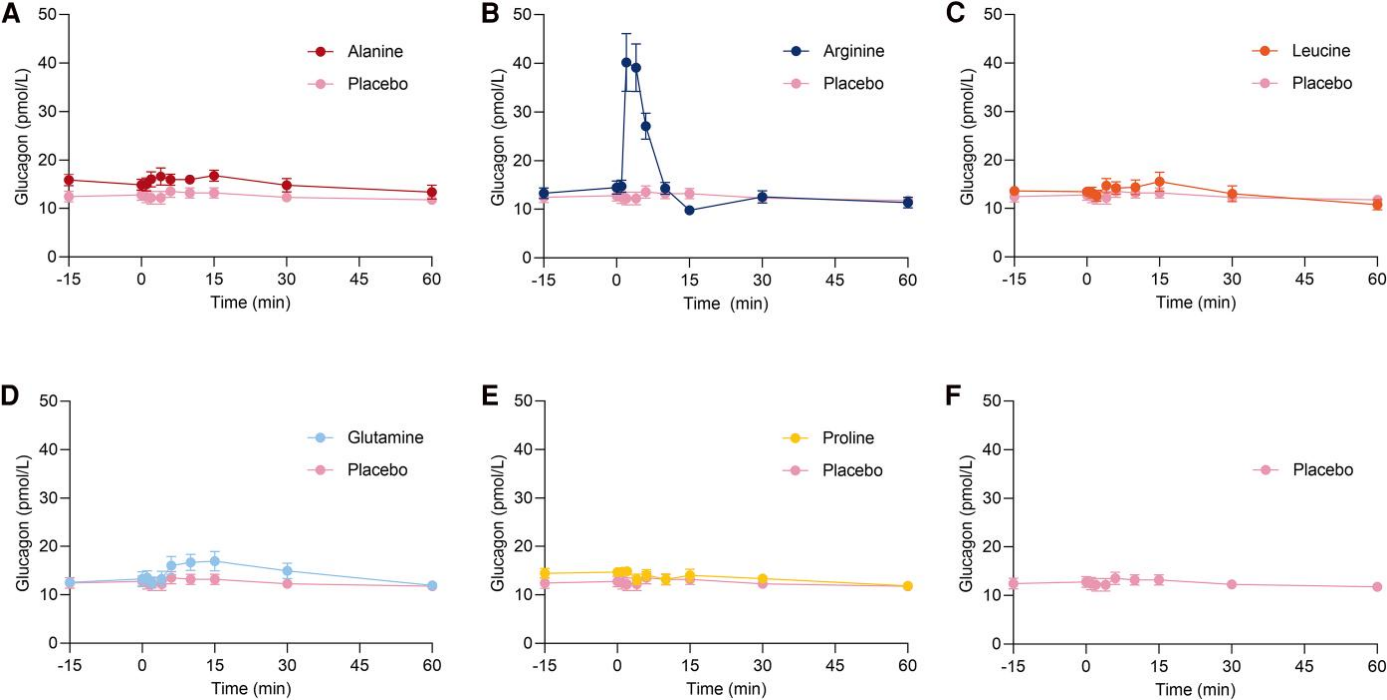
Plasma concentrations of glucagon after bolus injection of alanine (A), arginine (B), leucine (C), glutamine (D), and proline (E), compared to saline (placebo) (F) administered at time 0 minutes in 12 healthy male individuals on 6 separate experimental days, conducted in a double-blind, randomized order. Data are presented as mean values ± SEM.

The primary endpoint, iAUC_30_ for glucagon, was significantly larger after bolus injection of arginine compared to placebo by a mean of 99 [58; 140] pmol/L × min (*P* < .01). We also observed a significantly larger iAUC_60_ and iAUC_15_ after injection of arginine compared to placebo. Likewise, C_max_ for glucagon was significantly increased after injection of arginine by a mean of 29 [19; 40] pmol/L (*P* < .01). The iAUC_30_ for glucagon after injection of glutamine trended to be larger compared to placebo, though this difference was not significant (*P* = .063), and there was no significant difference from placebo after alanine injection (*P* = .25). C_max_ for glucagon was increased after injection of alanine by a mean of 4 [1; 6] pmol/L (*P* = .017). Notably, the fasting concentration of glucagon appeared higher compared to placebo on the day of alanine injection, although this was not significant (Fig. 2A). Injections of leucine and proline did not alter glucagon secretion. Data are presented in Fig. 2 and Table 2.

**Table 2.** Fasting conc., C_max_, and iAUC_15_, iAUC_30_,iAUC_60_ for glucagon, insulin, and C-peptide in 12 healthy male individuals after bolus injections of alanine, arginine, leucine, glutamine, proline, and saline (placebo), respectively, on 6 separate experimental days, conducted in a double-blind, randomized order.

		Placebo	Alanine			Arginine			Leucine			Glutamine			Proline		
		Mean ± SD	Est. diff. [95% CI]	*P*	Adj. *P*	Est. diff. [95% CI]	*P*	Adj. *P*	Est. diff. [95% CI]	*P*	Adj. *P*	Est. diff. [95% CI]	*P*	Adj. *P*	Est. diff. [95% CI]	*P*	Adj. *P*
**Glucagon**																	
Fasting conc. (pmol/L)		13 ± 4	2 [0; 4]	.052	.18	2 [−1; 4]	.19	.40	1 [−2; 3]	.56	.74	1 [−2; 3]	.69	.84	2 [1; 3]	<.01	<.05
C_max_ (pmol/L)		15 ± 4	4 [1; 6]	<.01	<.05	29 [19; 40]	<.0001	<.01	2 [0; 5]	.091	.18	3 [0; 6]	.067	.15	1 [0; 3]	.14	.21
iAUC_15_	pmol/L × min	19 ± 21	9 [−6; 25]	.21	.44	111 [70; 151]	<.0001	<.001	6 [−12; 24]	.48	.71	22 [−2; 47]	.070	.22	−10 [−28; 6]	.20	.41
iAUC_30_		34 ± 34	21 [−9; 51]	.15	.25	99 [58; 140]	<.001	<.01	20 [−23; 62]	.33	.39	54 [−4; 111]	.063	.16	−14 [−49; 21]	.39	.39
iAUC_60_		52 ± 50	42 [−17; 103]	.14	.21	90 [40; 140]	<.01	<.05	20 [−53; 94]	.55	.56	82 [−20; 189]	.11	.19	−17 [−76; 43]	.55	.56
**Insulin**																	
Fasting conc. (pmol/L)		42 ± 26	9 [−19; 37]	.49	.71	−9 [−24; 6]	.22	.46	−6 [−22; 11]	.46	.69	−8 [−17; 2]	.098	.27	−1 [−8; 5]	.62	.79
C_max_ (pmol/L)		51 ± 29	23 [−4; 51]	.091	.26	135 [90; 180]	<.0001	<.001	4 [−13; 22]	.61	.78	39 [22; 55]	<.001	<.01	−1 [−7; 6]	.86	.91
iAUC_15_	pmol/L × min	43 ± 49	77 [27; 126]	<.01	<.05	605 [384; 826]	<.0001	<.001	68 [16; 119]	.014	.058	291 [194; 388]	<.0001	<.001	10 [−28; 48]	.57	.75
iAUC_30_		93 ± 137	91 [−16; 197]	.089	.25	560 [322; 798]	<.001	<.01	55 [−21; 131]	.14	.34	341 [210; 473]	<.001	<.001	−18 [−100; 63]	**.63**	**.79**
iAUC_60_		152 ± 255	130 [−45; 304]	.13	.32	519 [229; 810]	<.01	<.05	18 [−112; 149]	.77	.87	333 [149; 517]	<.01	<.05	−26 [−191; 138]	**.73**	**.86**
**C-peptide**																	
Fasting conc. (pmol/L)		472 ± 223	56 [−128; 240]	.52	.73	−82 [−195; 32]	.14	.34	−35 [−153; 83]	.53	.74	−64 [−139; 10]	.084	.24	−35 [−69; 0]	**.049**	**.17**
C_max_ (pmol/L)		507 ± 234	104 [−83; 292]	.25	.48	336 [158; 515]	<.01	<.01	0 [−125; 124]	.99	.99	105 [5; 205]	.042	.15	−35 [−86; 15]	.15	.35
iAUC_15_	pmol/L × min	178 ± 158	402 [178; 625]	<.01	<.05	2956 [2007; 3905]	<.0001	<.001	261 [14; 507]	.040	.15	1191 [730; 1652]	<.001	<.001	23 [−151; 196]	.78	.88
iAUC_30_		344 ± 361	682 [180; 1184]	<.05	.051	3243 [1990; 4495]	<.001	<.001	320 [−91; 731]	.11	.30	2137 [1231; 3043]	<.001	<.01	−8 [−321; 305]	.96	.97
iAUC_60_		539 ± 636	979 [77; 1880]	.036	.14	3239 [1785; 4692]	<.001	<.01	293 [−291; 806]	.23	.47	2544 [1269; 3819]	<.01	<.01	9 [−496; 515]	.97	.98

Estimated differences are mean differences for approximately normally distributed data compared to placebo. *P*-values were adjusted to control the false discovery rate.

Abbreviations: CI, confidence interval; C_max_, maximum concentration; fasting conc., fasting concentration; iAUC_15_, incremental area under the curve for the first 15 minutes; iAUC_30_ incremental area under the curve for the first 30 minutes; iAUC_60,_ incremental area under the curve for the total 60 minutes.

### Alanine, Arginine, and Glutamine Exert Insulinotropic Effects After Bolus Injection

**Figure 3.**
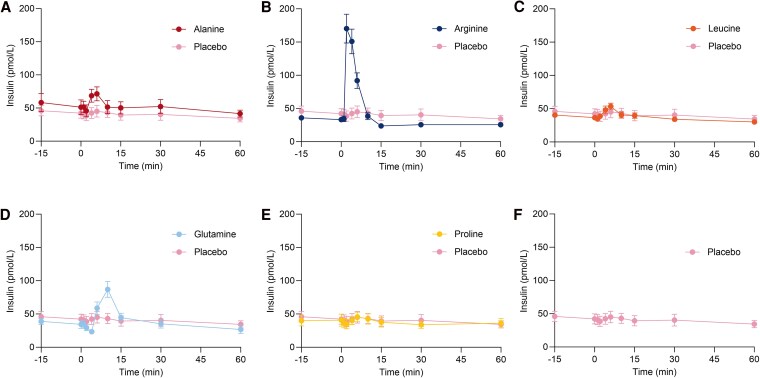
Plasma concentrations of insulin after bolus injection of alanine (A), arginine (B), leucine (C), glutamine (D), and proline (E) compared to saline (placebo) (F) administered at time 0 minutes in 12 healthy male individuals on 6 separate experimental days, conducted in a double-blind, randomized order. Data are presented as mean values ± SEM.

**Figure 4.**
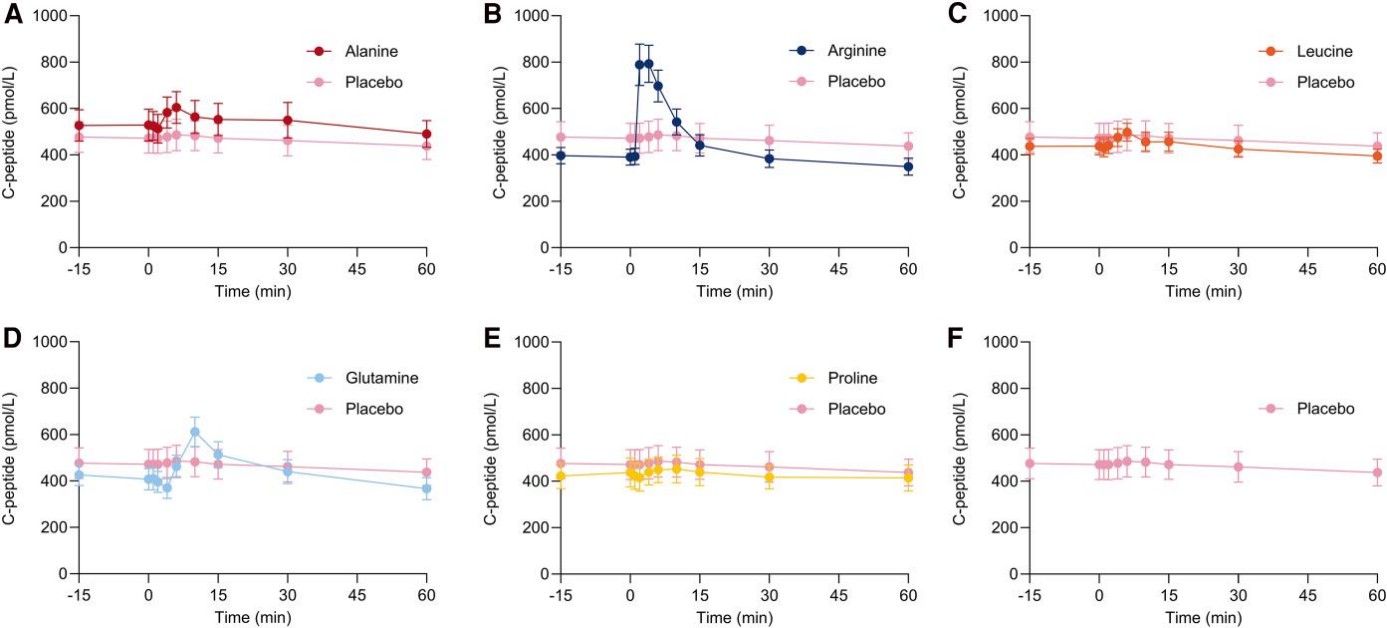
Plasma concentrations of C-peptide after bolus injection of alanine (A), arginine (B), leucine (C), glutamine (D), and proline (E) compared to saline (placebo) (F) administered at time 0 minutes in 12 healthy male individuals on 6 separate experimental days, conducted in a double-blind, randomized order. Data are presented as mean values ± SEM.

The iAUC_15_ for insulin was larger after injection of alanine (77 [27; 126] pmol/L × min, *P* < .05), arginine (605 [384; 826] pmol/L × min, *P* < .001), and glutamine (291 [194; 388] pmol/L × min, *P* < .001) compared to placebo. The iAUC_30_ and iAUC_60_ for insulin after injections of arginine and glutamine were significantly larger compared to placebo. The iAUC_15_ for insulin after injection of leucine was larger compared to placebo by a mean of 68 [16; 119] pmol/L × min, although this was not significant after adjusting for multiple testing. C_max_ for insulin was increased after injections of arginine (135 [90; 180] pmol/L, *P* < .001) and glutamine (39 [22; 55] pmol/L, *P* < .01). The C-peptide responses paralleled those of insulin. Data are presented in Figs. 3 and 4 and Table 2.

### Glucose and Incretin Hormones Are Unaffected by Amino Acid Injections

There were no changes in plasma glucose in response to the injections of amino acids or placebo (Supplemental Fig. S2 [29]). There appeared to be a slight increase in GLP-1 after injection of arginine, but this was driven by a single outlier and was not different from GLP-1 concentrations after injection of placebo (Supplemental Fig. S3B [29]). There were no changes in GLP-1 concentrations after injections of alanine, leucine, glutamine, or proline (Supplemental Fig. S3 [29]). Injection of proline led to a small and transient increase in C_max_ for GIP by a mean of 4 [1; 6] pmol/L (Supplemental Fig. S4E [29]). However, this was not reflected by a larger iAUC for GIP (Table 3). Injections of alanine, arginine, leucine, and glutamine did not affect GIP concentrations. Data are presented in Supplemental Figs. S2 to S4 [29] and Table 3.

**Table 3.** Fasting conc., C_max_, AUC_30_, and AUC_60_ for glucose, GLP-1, and GIP in 12 healthy male individuals after bolus injections of alanine, arginine, leucine, glutamine, proline, and saline (placebo), respectively, on 6 separate experimental days, conducted in a double-blind, randomized order.

		Placebo	Alanine			Arginine			Leucine			Glutamine			Proline		
		Mean ± SD	Est. diff. [95% CI]	*P*	Adj. *P*	Est. diff. [95% CI]	*P*	Adj. *P*	Est. diff. [95% CI]	*P*	Adj. *P*	Est. diff. [95% CI]	*P*	Adj. *P*	Est. diff. [95% CI]	*P*	Adj. *P*
**Glucose**																	
Fasting conc. (mmol/L)		5.0 ± 0.4	0 [−0.4; 0.4]	.96	.97	−0.2 [−0.4; 0]	.049	.17	−0.1 [−0.4; 0.2]	.54	.74	−0.2 [−0.4; 0.1]	.18	.39	−0.2 [−0.4; 0.1]	.13	.34
C_max_ (mmol/L)		5.2 ± 0.3	0 [−0.2; 0.3]	.88	.93	0.3 [0; 0.5]	.044	.16	−0.1 [−0.3; 0]	.12	.31	0.1 [−0.1; 0.3]	.39	.62	−0.1 [−0.3; .1]	.15	.36
AUC_30_	mmol/L × min	151 ± 11	1 [−7; 9]	.76	.87	1 [−5; 7]	.73	.86	−4 [−11; 2]	.17	.38	−1 [−6; 5]	.84	.91	−4 [−8; 1]	.13	.32
AUC_60_		302 ± 21	1 [−14; 16]	.93	.95	−1 [−12; 9]	.77	.87	−8 [−20; 4]	.16	.37	−5 [−16; 6]	.35	.58	−7 [−15; 2]	.10	.28
**GLP-1**																	
Fasting conc. (pmol/L)		10 ± 2	1 [−2; 3]	.52	.74	−1 [−3; 2]	.64	.80	0 [−3; 2]	.88	.93	0 [−3; 1]	.46	.69	0 [−2; 1]	.68	.83
C_max_ (pmol/L)		12 ± 2	1 [−1; 3]	.54	.74	3 [−3; 9]	.32	.57	−1 [−3; 2]	.60	.77	0 [−2; 2]	.78	.88	−1 [−2; 0]	.078	.24
AUC_30_	pmol/L × min	289 ± 63	26 [−18; 71]	.23	.46	4 [−34; 42]	.83	.90	4 [−59; 66]	.90	.94	9 [−24; 42]	.55	.74	−23 [−59; 13]	.19	.41
AUC_60_		572 ± 126	24 [−71; 119]	.60	.77	−17 [−89; 54]	.60	.78	−14 [−144; 116]	.82	.89	23 [−67; 113]	.58	.77	−59 [−114; −5]	.034	.13
**GIP**																	
Fasting conc. (pmol/L)		13 ± 3	3 [−2; 8]	.23	.46	0 [−3; 3]	.82	.89	1 [−2; 4]	.53	.74	−2 [−4; 0]	.099	.27	3 [0; 5]	.068	.22
C_max_ (pmol/L)		16 ± 3	3 [−2; 8]	.17	.39	1 [−2; 3]	.59	.77	1 [−1; 4]	.27	.51	−1 [−3; 0]	.10	.27	4 [1; 6]	<.01	<.05
AUC_30_	pmol/L × min	408 ± 86	−18 [−78; 42]	.52	.74	−7 [−123; 109]	.90	.94	−15 [−109; 80]	.74	.86	36 [−29; 101]	.25	.49	−45 [−139; 50]	.25	.49
AUC_60_		808 ± 171	−82 [−240; 75]	.27	.52	−7 [−235; 221]	.095	.96	−94 [−317; 129]	.43	.66	−53 [−88; 193]	.43	.66	−163 [−345; 20]	.076	.24

Estimated differences are mean differences for approximately normally distributed data compared to placebo. *P*-values were adjusted to control the false discovery rate.

Abbreviations: AUC_30_, area under the curve for the first 30 minutes; AUC_60_, area under the curve for the total 60 minutes; C_max_, maximum concentration; fasting conc., fasting concentration; GIP, glucose-dependent insulinotropic polypeptide; GLP-1, glucagon-like peptide.

## Discussion

We investigated the effects of equimolar bolus injections of alanine, arginine, leucine, glutamine, and proline on glucagon secretion in healthy males. Under the conditions of our study, we observed that arginine significantly increased plasma glucagon concentrations, while a minor increase was observed for alanine, and glutamine exhibited a trend toward elevation. We also show that arginine, alanine, and glutamine increased insulin secretion while the secretion of incretin hormones was generally unaffected by the injections.

The present findings are consistent with previous observations suggesting that arginine is the most glucagonotropic amino acid in humans [5, 17, 26, 33-36]. Few studies have systematically compared the glucagonotropic effects of amino acids in animals [4, 5, 25, 26], and similar research in humans has been lacking. Although arginine consistently has been shown to stimulate glucagon secretion, it was not the most potent amino acid in all preclinical studies [4, 5, 25]. In our study, bolus injection of arginine at a dose equivalent to 20% of that used in the arginine test elicited an immediate and significant increase in glucagon levels (Fig. 2 and Table 2). Alanine has been identified as 1 of the most potent stimulators of glucagon secretion in both sheep and perfused mouse pancreas models [5, 26]. Given the association between alanine and glucagon action, it has been hypothesized that alanine plays a predominant role in regulating glucagon secretion [15, 16, 37]. In the present study, the iAUC for glucagon after injection of alanine was not increased, but there was a small increase in C_max_ of glucagon (Fig. 2 and Table 2). On the days of alanine injection, the fasting concentration of glucagon appeared higher compared to placebo days, but this was not significant (Fig. 2A and Table 2). In light of previous studies demonstrating that alanine stimulates glucagon secretion in humans [24, 38-42], our finding of a limited glucagon response to alanine injection was unexpected. It is important to note that previous studies used higher alanine doses than those in the present study, resulting in 6- to 20-fold increases in plasma alanine levels, substantially exceeding the fold change observed in our study [24, 39, 40]. The glucagon response to the administration of arginine is known to be dose-dependent [36], and this is likely also true for alanine. Thus, the lower dose of alanine used in the present study may explain the discrepancy in its glucagonotropic effect reported here compared to previous findings. Alanine concentrations have been reported to increase 1.2- to 1.7-fold after a protein-rich meal [43-45]. In our study, alanine levels increased 2.6-fold, indicating that we reached supraphysiological concentrations. Similarly, arginine levels, which increase 1.1- to 3-fold postprandially [43-45], increased 8.6-fold in our study. These discrepancies potentially influenced the observed differences in glucagon secretion as amino acids with high basal concentrations may require greater absolute increases to elicit similar effects. The varying fold increases in the concentrations of the injected amino acids represent a limitation of our study and complicate direct comparisons of their glucagonotropic effects.

To determine a suitable dose of amino acids, we considered administering each amino acid in proportion to its fasting plasma concentration. However, because fasting plasma levels of the selected amino acids range from 100 to 800 μmol/L, it would have been difficult to distinguish whether differences in their glucagonotropic effects were caused by inherent potency or simply differences in dosing. We therefore opted to administer the amino acids at equimolar doses, consistent with the methodology employed in preclinical studies, although much higher doses were used in those studies [4, 5]. Therefore, our results reflect the dose relationship, which is not necessarily equivalent to physiologic potency.

We were unable to measure an increase in glutamine following its injection, raising the possibility that it was rapidly taken up by the liver and metabolized to glutamic acid. However, glutamic acid levels also remained unchanged (data not shown), suggesting that other methodological limitations or metabolic factors may explain the undetectable glutamine increase. To rule out issues with the administered compound, we confirmed that the vials used in the study contained L-glutamine (data not shown). Furthermore, we observed glutamine-induced insulin secretion, and the reported side effects of glutamine injection were consistent with those described after intraduodenal glutamine administration [46]. However, glutamine injection did not stimulate glucagon secretion, aligning with findings from a study using the perfused mouse pancreas [26].

Studies in mouse models with disrupted glucagon signaling (ie, glucagon receptor knockout or glucagon receptor antagonist treatment) have suggested that both glutamine and alanine are important regulators of glucagon secretion by stimulating α cell proliferation [14, 15]. Arginine has also been suggested as an important regulator of α cell proliferation and glucagon secretion. In line with this, hepatic expression of the arginine transporter *SLC7A2* was downregulated in mice after disruption of glucagon signaling, and arginine levels were increased [14, 15]. Similarly, we have shown that the hepatic expression of *SLC7A2* was downregulated in individuals with obesity and glucagon resistance to amino acid catabolism, although fasting arginine concentrations were similar compared to lean individuals [47]. These studies indicate that arginine could be an important regulator of the liver-α cell axis, and our finding that arginine efficiently stimulates glucagon secretion supports this.

Proline has also been suggested as an important regulator of glucagon secretion [26]. Fasting proline concentrations were shown to be increased in humans with glucagon resistance [47, 48], but the injection of proline in the present study did not stimulate glucagon secretion (Fig. 2 and Table 2). However, the isolated increase in 1 amino acid does not reflect physiologic conditions, and it has been suggested that α cell function is determined by the integration of the effects of amino acids, glucose, and fatty acids [37]. Hormone levels may also influence this relationship, as GIP has been shown to potentiate the glucagonotropic effect of amino acids in mice [49].

In 1966, it was demonstrated that both the ingestion of protein and the IV administration of a mixture of amino acids stimulate insulin secretion in humans [50, 51]. Among the 10 amino acids tested, arginine was found to most potently stimulate insulin secretion [51]. In the present study, we observed that arginine, glutamine, and alanine all stimulated insulin secretion, with arginine being the most potent (Fig. 3 and Table 2). Conversely, proline injection did not stimulate insulin secretion, while leucine showed a trend toward stimulation. The lack of a significant response to leucine in the present study may be due to a lower dose compared to previous studies [3-5]. Leucine has been demonstrated to increase 2.2- to 3.3-fold after a protein-rich meal [43, 44]. In our study, leucine increased 4.6-fold, again indicating that we reached supraphysiological concentrations. Leucine and glucose have a synergistic effect on insulin secretion [21], suggesting that the effect of leucine might differ in the context of a mixed meal. As in previous studies, leucine did not increase glucagon secretion [4, 5]. Given the importance of leucine for muscle protein synthesis, it seems advantageous that leucine stimulates anabolism by promoting insulin secretion without the catabolic effects of glucagon, and leucine likely promotes insulin secretion by acting directly on the β cell [5, 52]. In contrast, arginine may stimulate insulin secretion by acting both directly on the β cells and indirectly by stimulating glucagon secretion [53]. Supporting the latter, a study using perfused mouse islets showed that arginine and glutamine failed to stimulate insulin secretion in the absence of both glucagon and GLP-1 signaling, and in isolated perfused mouse pancreas, increased glucagon secretion contributed importantly to amino acid-stimulated insulin secretion [54, 55]. In the present study, insulin secretion was stimulated by these amino acids without a concomitant increase in incretin hormones, indicating that their effects are not dependent on a rise in GLP-1 concentrations.

In conclusion, in a direct comparison of equimolar IV bolus injections of alanine, arginine, leucine, glutamine, and proline, arginine most potently stimulated glucagon secretion compared to placebo in healthy male volunteers. Injections of alanine, arginine, and glutamine stimulated insulin secretion, with arginine being the most potent. Our findings indicate that arginine is a potential regulator of the liver-α cell axis, but they do not exclude the potential regulatory role of other amino acids.

## Data Availability

Some or all datasets generated during and/or analyzed during the current study are not publicly available but are available from the corresponding author upon reasonable request.

## Clinical Trial Information

The study was registered with www.clinicaltrials.gov (ID: NCT05954923).

## Abbreviations

arginine-HCl: arginine-hydrochloride
C_max_: maximum concentration
GIP: glucose-dependent insulinotropic polypeptide
GLP-1: glucagon-like peptide 1
iAUC: incremental area under the curve
